# An Insight into the Increasing Role of LncRNAs in the Pathogenesis of Gliomas

**DOI:** 10.3389/fnmol.2017.00053

**Published:** 2017-02-28

**Authors:** Yuanliang Yan, Zhijie Xu, Zhi Li, Lunquan Sun, Zhicheng Gong

**Affiliations:** ^1^Department of Pharmacy, Xiangya Hospital, Central South UniversityChangsha, China; ^2^Institute of Hospital Pharmacy, Central South UniversityChangsha, China; ^3^Department of Pathology, Xiangya Hospital, Central South UniversityChangsha, China; ^4^Center for Molecular Medicine, Xiangya Hospital, Key Laboratory of Molecular Radiation Oncology of Hunan Province, Central South UniversityChangsha, China

**Keywords:** glioma, lncRNAs, diagnostic biomarkers, therapeutic targets, therapy

## Abstract

Long non-coding RNAs (LncRNAs) are essential epigenetic regulators with critical roles in tumor initiation and malignant progression. However, the roles and mechanisms of aberrantly expressed lncRNAs in the pathogenesis of gliomas are not fully understood. With the development of deep sequencing analyses, an extensive amount of functional non-coding RNAs has been discovered in glioma tissues and cell lines. Additionally, the contributions of several lncRNAs, such as Hox transcript antisense intergenic RNA, H19 and Colorectal neoplasia differentially expressed, previously reported to be involved in other pathogenesis and processes to the oncogenesis of glioblastoma are currently addressed. Thus, lncRNAs detected in tumor tissues could serve as candidate diagnostic biomarkers and therapeutic targets for gliomas. To understand the potential function of lncRNAs in gliomas, in this review, we briefly describe the profile of lncRNAs in human glioma research and therapy. Then, we discuss the individual lncRNA that has been under intensive investigation in glioma research, and the focus is its mechanism and clinical implication.

## Introduction

Glioblastoma (GBM) is the most common primary intracranial tumor, with varying malignancy grades and histological subtypes. Although relatively rare in occurrence, GBM frequently causes mortality and morbidity (Ostrom et al., 2014; Bian et al., 2015), and its median survival time is only 12–14 months after initial diagnosis (Stetson et al., 2016). The current standard therapy for GBM is concomitant radiochemotherapy following maximal surgical tumor resection. However, aggressive growth and recurrence frequently follows after the optimal treatment (Penaranda Fajardo et al., 2016). It is conceivable that complicated signaling pathways and related molecular events underlie the development of gliomas. Consequently, investigations exploring the accurate molecular mechanisms and reliable therapeutic targets for GBM have drawn extensive attention and provided a hopeful prospect for GBM treatment (Kitambi et al., 2014; Furnari et al., 2015).

**FIGURE 1:**
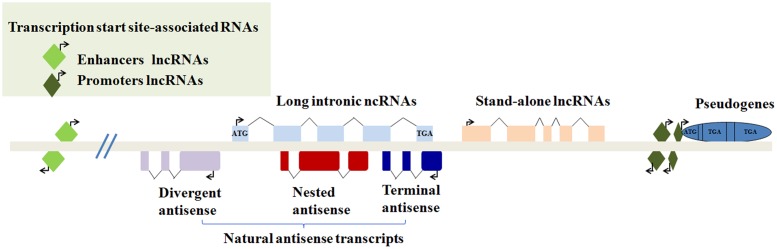
**Classification of lncRNAs according to their genomic location.** (1) Transcription start site-associated RNAs may be transcribed from enhancers or promoters. (2) Long intronic ncRNAs may be transcribed from introns of other genes. (3) Natural antisense transcripts contain divergent antisense, nested antisense or terminal antisense. (4) Stand-alone transcription units. (5) Some transcribed pseudogenes.

Recently, epigenetic regulation has also drawn remarkable attention, particularly in terms of lncRNAs, which are indispensable for the regulation of cellular processes. LncRNAs are transcripts of more than 200 nucleotides without functional protein-coding ability in a conventional way (Quinn and Chang, 2016). Intriguingly, their coding and translation potential have been reported; they may act as a repository for the synthesis of small polypeptides with interesting biological activity (Cohen, 2014; Ruiz-Orera et al., 2014). LncRNAs can be grouped into five non-exclusive categories according to their genomic location. The subcellular localization is a good indication of the putative function of a lncRNA (Schmitz et al., 2016) (**Figure 1**). For the past three decades, lncRNAs have been found to regulate gene expression during both biological and pathological processes (Fatica and Bozzoni, 2014). For instance, lncRNAs can work as cellular “address codes,” which allows protein complexes to be transferred to the appropriate locations on chromosomes and results in consequent activation or deactivation (Batista and Chang, 2013). Mechanistically, in contrast to small interfering RNAs (siRNAs) and microRNAs, lncRNAs can fold into higher order structures to provide much greater potential for target recognition, which facilitates chromatin remodeling as well as transcriptional and post-transcriptional regulation (Mercer and Mattick, 2013; Sahu et al., 2015).

In accordance with their significant roles in normal biological processes, lncRNAs have been implicated in the oncogenesis of gliomas and are increasingly being considered potential therapeutic targets (Ma et al., 2016; Schmitt and Chang, 2016). For example, the well-studied HOTAIR, a lncRNA highly expressed in breast cancer that participates mainly in the chromatin remodeling process, was found to be associated with the biogenesis, development and differentiation of gliomas (Bian et al., 2016). Furthermore, some newly discovered lncRNAs have been found in glioma tissue and cell lines, such as lncRNA ASLNC22381and KIAA0495 (Trojan et al., 2003; Zhang X.Q. et al., 2013, 2015). Through investigating the lncRNAs in tissue specimens for their expression stability in human gliomas and normal brain, Kraus et al. (2015) identified four lncRNAs (HOXA6as, H19 upstream conserved 1 and 2, Zfhx2as and BC200) with stable expression levels in gliomas compared with normal brain. Collectively, these lncRNAs have gained value for clinical purposes as novel biomarkers, but despite this great potential, many issues remain in this rapidly growing field. Here, we summarize the most up-to-date findings regarding how lncRNAs are regulated at the molecular level and their implications in the areas of glioma research and therapy.

## Profile of LNCRNAS in Human Glioma Research and Therapy

Recent studies in the large-scale analyses of full-length cDNA sequences have discovered many lncRNAs as key players of cell differentiation, immune responses, tumorigenesis, and other biological processes (Wakamatsu et al., 2009; Fujimoto et al., 2016; Wang J. et al., 2016). The Cancer Genome Atlas (TCGA), an ambitious and successful cancer genomics project, generates large-scale multi-dimensional genomic datasets covering over 20 malignancies, providing valuable insights into the underlying genetic and genomic alteration of cancer (Wang Z. et al., 2016). Deep sequencing studies, including large consortia, such as TCGA, have identified numerous tumor-specific mutations not only in protein-coding sequences, but also in non-coding sequences, which have proven to be an important component hidden in the “dark matter” of the genome. These cancer-associated mutations within non-coding RNA, including lncRNAs, can affect gene regulation in the pathogenesis and development of gliomas (Ramos et al., 2016; Diederichs et al., 2016). Differentially expressed lncRNAs in gliomas have been widely analyzed using human glioma tissues and cell lines (**Table 1**). These studies indicated that abnormal lncRNAs plays critical roles in the development and progression of gliomas.

**Table 1:** Long non-coding RNAs (lncRNAs) expression profile in glioma tissues and cell lines.

Samples	Target gene-related pathways	GEO item	Key lncRNA-mRNA	LncRNA expression	Reference
1 GBM tissue vs. 1 normal tissue	PPAR signaling pathway	NA	ASLNC22381-IGF-1, ASLNC20819-IGF-1	Up-regulated	Han et al., 2012
268 GBM tissue vs. 8 normal tissue	NA	NA	C21orf131-B, MEG3, RFPL1S	Down-regulated	Zhang X. et al., 2012


			CRNDE, HOTAIRM1	up-regulated	
213 GBM tissue	NA	GSE7696, GSE16011	PART1, MGC21881, MIAT, GAS5, PAR5	Longer survival	Zhang X.Q. et al., 2013


			KIAA0495	shorter survival	
475 samples	IDH1 mutation, 1p/19q LOH, EGFR amplification	GSE16011	LncR1	Poor prognosis	Li R. et al., 2014


			LncR3	better prognosis	
510 GBM tissue vs. 374 normal tissue	Pluripotent stem cells	NA	uc.283-plus	Up-regulated	Galasso et al., 2014
5 GBM tissue vs. 5 normal tissue	DNA replication, cell signaling, RNA degradation	NA	AC092168.4, RP11-90M5.4, LOC285768, BC105019, AC133528.2, AC013472.6, AL031123.1, AC022311.1	Up-regulated	Yan et al., 2015
			RP11-23B15.1, AK026168, RP11-439L18.3	Down-regulated	
SHG-139 cells vs. stem cells SHG-139S	NA	NA	CUST-64397-P1427979520, CUST-3094-P142797520, CUST-33806-P1427979520	Down-regulated	Li Y. et al., 2015
			CUST-7128-P1427979520, A23 P103812	Up-regulated	
3 pair of primary and recurrent gliomas	Systemic lupus erythematosus, antigen processing and presentation, FoxO signaling pathway, GnRH signaling pathway, ErbB signaling pathway, MAPK signaling pathway	Biological process, cellular component, molecular function	RP5-998N21.4, RP11-196G18.3, MGC32805, RP11-439A17.9, ADAMTS9-AS1, RP4-792G4.2	Up-regulated	Chen et al., 2015
			XLOC_000669, TUBA4B	Down-regulated	
U251 and U87cells	NA	NA	MEG3, ST7OT1, GAS5	up-regulated with DOX	Liu Q. et al., 2015
			MEG3, ST7OT1, neat1, MIR155HG	up-regulated with Rsv	
			TUG1, BC200, MIR155HG	Down-regulated with DOX	
GBM samples with mutant-type and wild-type IDH1	Metabolism, cell growth,	NA	KIAA0495, RP11-38P22.2, HOTAIRM1	down-regulated	Zhang X.Q. et al., 2015


	apoptosis		LOC254559, LINC00689	up-regulated	


*DOX, doxorubicin; Rsv, resveratrol; NA, not available.*

The lncRNA profile in clinical specimens reveals their potential roles in GBM pathogenesis. Using microarrays to analyze the tissues of GBM patients and age-matched normal donors, Han et al. (2012) found the lncRNA expression profile in GBM tissue is significantly altered. In GBM tissue, 654 lncRNAs are up-regulated (fold change ≥4.0), and 654 are down-regulated (fold change ≤0.25). Among the up-regulated lncRNAs, ASLNC22381 and ASLNC2081 are likely to serve as the key elements in the regulation of glioma signaling pathways. Target gene-related pathway analysis indicated that ASLNC22381 and ASLNC20819 may play important roles via their target insulin-like growth factor 1 (IGF-1) genes, which has been thought to be a positive risk factor for human glioma development (Rohrmann et al., 2011). In addition, applying the Affymetrix HG-U133 Plus 2.0 array, Zhang X. et al. (2012) revealed that in tumors relative to normal brain tissues, lncRNA C21orf131-B, MEG3, and RFPL1S are down-regulated, while HOTAIRM1 (HOX antisense intergenic RNA myeloid 1) and CRNDE are comparably up-regulated. Of note, these lncRNA expression patterns show a close correlation with malignancy grade and histological differentiation in human gliomas (Zhang X. et al., 2012). The same group later identified a set of six lncRNAs in 107 GBM patients, including KIAA0495, PART1, MGC21881, MIAT, GAS5, and PAR5, that are significantly associated with overall survival. The prognostic value of this six-lncRNA signature is independent of the methylation status of O-6-methylguanine-DNA methyltransferase (MGMT) promoter, which can promote the treatment resistance of glioma cells to alkylating agent chemotherapy (Zhang X.Q. et al., 2013; Wick et al., 2014). Moreover, based on the lncRNA expression profiles, Li R. et al. (2014) identified three novel molecular subtypes (named LncR1, LncR2 and LncR3) in gliomas. Survival analysis indicated that the LncR1 subtype has the poorest prognosis, while the LncR3 subtype shows the best overall survival rate (Li R. et al., 2014). Another study on lncRNA and mRNA interactions revealed that lncRNAs, such as Hox cluster-associated lncRNAs, can modulate a list of genes participating in the pathogenesis of GBM (Yan et al., 2015). In addition, the expression profiles analysis in recurrent gliomas compared with primary gliomas identified abundant differentially expressed lncRNAs, such as H19, CRNDE, and HOTAIRM1. These results imply that the future studies of specific expressed lncRNAs would help elucidate the mechanism of glioma recurrence at the genetic level and identify effective therapeutic targets for glioma patients (Chen et al., 2015).

Additionally, *in vitro* studies have strongly suggested that the altered expression of lncRNAs during genome mutation or genotoxic stress is involved in multiple neuro-oncological disorder-associated cellular processes. Isocitrate dehydrogenase 1 (IDH1) mutations have been shown to be an important prognostic marker for patients with gliomas (Cai et al., 2016; Wang P.F. et al., 2016). LncRNA profiling between gliomas with or without IDH1 mutations show significantly altered gene expressions in astrocytic and oligodendroglial tumors. Among the differential lncRNAs, KIAA0495, LOC254559 and LOC255130 have a close correlation with clinical outcomes in IDH1-mutant patients. Moreover, these three IDH1 mutation-associated lncRNAs participate in multiple tumor-associated cellular biological behaviors, including cell proliferation, apoptosis and metastasis (Zhang X.Q. et al., 2015). In addition, after treatment with DNA damaging reagents, such as doxorubicin and resveratrol, specific candidate lncRNAs (MEG3, ST7OT1, TUG1, BC200 and MIR155HG) are detected in human glioma cell lines (U251 and U87). During apoptosis induced by both reagents, MEG3 and ST7OT1 are up-regulated in both cell lines. Instead, when necrosis is induced with a high dose of doxorubicin, TUG1, BC200 and MIR155HG are significantly down-regulated (Liu Q. et al., 2015). As NEAT2 (nuclear-enriched abundant transcript 2), also known as MALAT1 (metastasis-associated lung adenocarcinoma transcript 1), is a highly conserved lncRNA associated with the metastatic potential of tumor cells, Han et al. (2016a) found that the knockdown of NEAT2 by RNA interference could promote the invasion and proliferation of glioma cells. Concomitantly, the apoptosis rate of the glioma cell lines is shown to dramatically increase (Han et al., 2016a; Xiang et al., 2016). Over all, these results indicate that an investigation into the abnormal expression profiles of lncRNAs may help in the understanding of oncogenesis and identify novel potential treatment targets in glioma research and therapy.

Accumulating evidence indicates that a rare population of self-renewing cells, called tumorigenic CSCs, is responsible for tumor formation and therapeutic resistance in gliomas (Lathia et al., 2015). Studies have indicated that lncRNAs are involved in several biological processes in CSCs (Li Y. et al., 2015). A large-scale expression study of functional ultra-conserved (uc) ncRNAs showed that the uc.283 lncRNA, a 277 nucleotide-long sequence located at ultra-conserved regions (UCRs) of human genes, is highly specific for pluripotent stem cells, as well as some solid cancers, particularly gliomas (Galasso et al., 2014). Moreover, Han et al. (2016b) found that the down-regulation of NEAT2 suppresses the expression of stemness markers Sox2 and Nestin, and further promotes cell proliferation by regulating the ERK/MAPK (extracellular signal-regulated kinase/mitogen-activated protein kinase) signaling axis in the glioma stem cell line SHG139. Furthermore, the knockdown of the lncRNA XIST could exert tumor-suppressive effects in human GBM stem cells by up-regulating miR-152 (Yao et al., 2015). In addition, as the gene enhancer of zeste homolog 2 (EZH2) serves as an oncogene and is required for cancer stem cell maintenance, the inhibition of EZH2 by lncRNAs can effectively promote the therapeutic sensitivity in gliomas (van Vlerken et al., 2013; Yin et al., 2016). Based on these observations, much more attention should be paid on the regulation of lncRNAs in the maintenance of glioma stem cells (GSCs), a decisive event occurring in the development of gliomas.

## Aberrantly Expressed LNCRNAS and Their Implications in Human Gliomas

**FIGURE 2:**
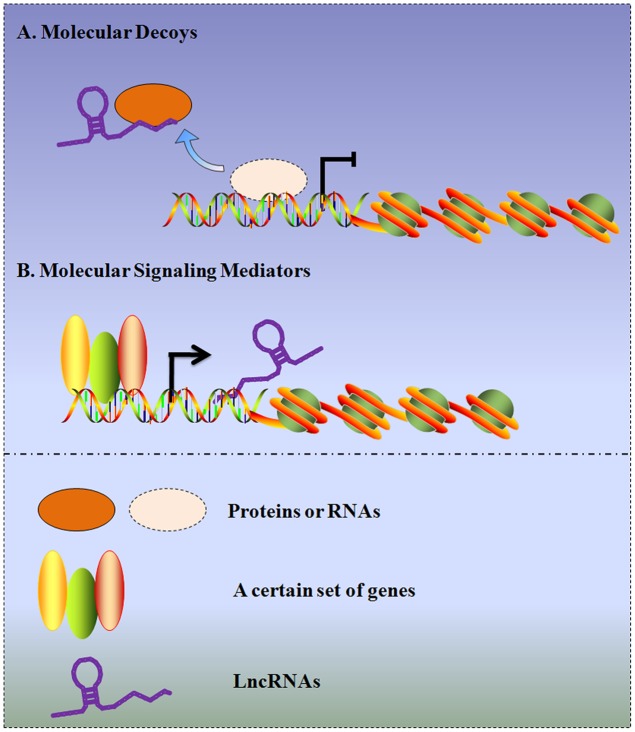
**Schematic diagram of the two archetypes of lncRNA mechanisms in gliomas.** **(A)** LncRNAs in gliomas can serve as molecular decoys, which take proteins or RNAs away from a specific location. **(B)** LncRNAs in gliomas can serve as molecular signaling mediators, which modulate the expression of a certain set of genes.

The differential expression patterns of lncRNAs between tumor and normal tissues, along with the expression discrepancies in tumors with different clinical features, provide the possibility that lncRNAs act as diagnostic, prognostic biomarkers and pharmaceutical targets in gliomas. Although an increasing number of lncRNAs are being characterized, their detailed mechanisms are still not completely elucidated. In this regard, recent studies have demonstrated that lncRNAs in gliomas can serve as molecular decoys, which move proteins or RNAs away from a specific location, like a “sponge” to miRNAs (e.g., HOTAIR/miR-326, CASC2/miR-21, XIST/miR-152, and Gas5/miR-222). Additionally, other investigations demonstrate lncRNAs can function as molecular signaling mediators, which modulate the expression of a certain set of genes (e.g., H19/CD133 and NEAT2/MMP2) (**Figure 2**). To provide an exhaustive description of the rapid development in this field, the molecular mechanisms and potential functions of several representative lncRNAs in gliomas will be discussed in the following sections.

### LncRNA HOTAIR

Long non-coding RNA HOTAIR, transcribed from the antisense strand of the homeobox C (HOXC) gene locus in chromosome 12, is involved in the regulation of specific gene transcription. A study by Tsai et al. (2010) demonstrated that HOTAIR regulates gene expression by interacting with polycomb repressive complex 2 (PRC2) and lysine-specific demethylase 1A (LSD1). The 5′- and 3′-domains of HOTAIR can bind to the PRC2 and LSD1/CoREST/REST complex, respectively. Serving as a scaffold, HOTAIR can tether two distinct complexes together and recruit specific histone modification enzymes, thereby resulting in H3K27 methylation and H3K4 demethylation and ultimately gene silencing (Tsai et al., 2010). In addition, HOTAIR could also serve as an inducer of ubiquitin-mediated proteolysis to control protein levels. HOTAIR facilitates the ubiquitination of Ataxin-1 through E3 ubiquitin ligases Dzip3, Snurportin-1, and Mex3b to further accelerate their degradation. Through the rapid decay of targets Ataxin-1 and Snurportin-1, HOTAIR can prevent cellular premature senescence (Yoon et al., 2013). In addition, aberrant HOTAIR expression has been extensively revealed to correlate with cancer metastasis and is characterized as a negative prognostic factor for cancer patients (Cai et al., 2014; Wu et al., 2014).

**FIGURE 3:**
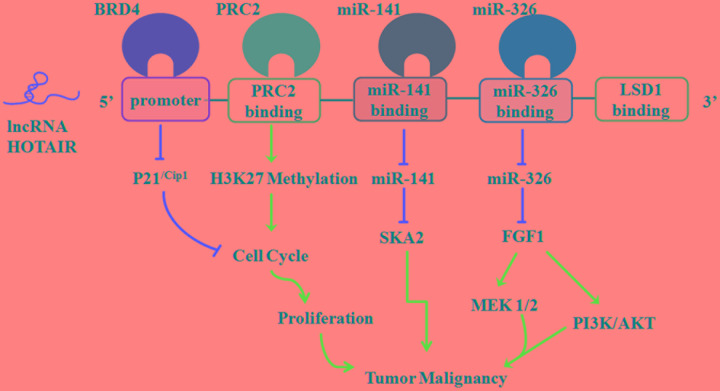
**Roles of lncRNA HOTAIR in glioma malignancy.** See text for detailed discussion.

Hox transcript antisense intergenic RNA expression is up-regulated in glioma tissues and cell lines, and can serve as a potential biomarker or therapeutic target for human gliomas (Kiang et al., 2015; Zhou et al., 2015) (**Figure 3**). Recent studies have indicated that HOTAIR expression is a critical regulator of cell cycle progression in gliomas (Zhang J.X. et al., 2013). HOTAIR regulates cell cycle progression predominantly via the HOTAIR 5′-domain-PRC2 axis, which is EZH2 (predominant PRC2 complex component)-dependent in GBM cells (Zhang K. et al., 2015). In addition, bromodomain and extraterminal (BET) domain proteins are required for GBM cell proliferation. BET protein inhibitors can reduce the proliferation of gliomas, in part, through the induction of the cyclin-dependent kinase inhibitor p21^Cip1^ *in vitro* and *in vivo* (Pastori et al., 2014). Pastori et al. (2014) found that the bromodomain protein BRD4 could directly control HOTAIR expression by binding to its promoter. The overexpression of HOTAIR in conjunction with the BET protein inhibitor I-BET151 abolishes the anti-proliferative activity of the BET bromodomain inhibitor (Pastori et al., 2015). Furthermore, the HOTAIR-miRNA axis has an important role in malignant biological behaviors of human glioma. Ke et al. (2015) found that fibroblast growth factor 1 (FGF1) mediates oncogenic effects by activating the PI3K/AKT and MEK 1/2 pathways. HOTAIR, one target of miR-326, has been confirmed to down-regulatemiR-326; then, it exerts its tumor-suppressive activities by reducing the expression of FGF1 (Ke et al., 2015). Similarly, HOTAIR can act as an endogenous “sponge” of miR-141, thereby promoting the promoter methylation of miR-141 by DNA methyltransferase 1 (DNMT1) in glioma cells. Then, the hypermethylated miR-141 can repress the expression of the spindle and kinetochore associated complex subunit 2 (SKA2), which results in a significant increase in tumor growth (Bian et al., 2016). Collectively, these results suggest that HOTAIR may potentiate glioma development in many facets; thus, it is worthy of further investigation.

### LncRNA H19

Long non-coding RNA H19, produced from the imprinted gene H19, is one of the most highly conserved transcripts involved in mammalian development. Studies have also demonstrated that H19 could potentially serve as an oncogenic lncRNA in different types of cancers, including gliomas (Kiang et al., 2015; Chen et al., 2016). Mechanistically, the product of the MYC oncogene, c-Myc, induces the expression of the H19 non-coding RNA, thereby potentiating gliomagenesis (Barsyte-Lovejoy et al., 2006). Furthermore, serving as a miRNA precursor, H19 could modulate glioma progression by generating miR-675. The oncogenic function of H19/miR-675 is dependent on the expression of cancer-associated cadherin 13 (CDH13), which is the direct target of miR-675 (Shi et al., 2014). Li C. et al. (2015) found that miRNA-675, which is derived from the first exon of H19, could regulate the immoderate proliferation and migration of glioma cell lines by inhibiting the expression of CDK6, which is a pivotal regulator of the cell cycle and involved in glioma development (Rader et al., 2013; Sherr et al., 2016). These findings agree with another study showing that H19 overexpression can promote the cell-cycle progression of cancer cells (Berteaux et al., 2005). Moreover, the knockdown of H19 by siRNA displays higher therapy efficiency when induced by the chemotherapy drug TMZ in GBM cells (Li W. et al., 2016). Thus, LncRNA H19 could be increasingly recognized as a potential target for glioma treatment.

Accumulating evidence has identified that tumorigenic CSCs, with self-renewing capability, contribute to tumor initiation and therapeutic resistance (Lathia et al., 2015). Intriguingly, H19 overexpression could maintain the stem cell properties of GBM cells. Li W. et al. (2016) found that the markers of CSCs, including CD133, NANOG, Oct4, and Sox2, are significantly down-regulated in H19-deficient cells. This conclusion was further confirmed by Jiang et al. (2016), who found that the increased level of H19 promotes invasion, angiogenesis, and stemness of GBM cells. H19 is significantly overexpressed in CD133-positive GBM cells, and higher H19 expression levels are associated with increased tumor growth (Jiang et al., 2016). In spite of the critical role of H19 in the maintenance of glioma stemness, its exact mechanism is still unclear and needs to be further investigated.

### LncRNA CRNDE

Colorectal neoplasia differentially expressed was initially identified by Derrien et al. (2012) as a putative non-coding RNA; it is highly expressed in developmental neurobiology and neuropathology. Studies have found that CRNDE expression is also elevated in many colorectal cancers and brain cancers, such as GBM, astroblastomas, and astrocytomas (Ellis et al., 2012; Kiang et al., 2015). Of note, among the 129 lncRNAs differentially expressed in glioma tissues, CRNDE is consistently identified as the most up-regulated lncRNA by 32-fold up (Zhang X. et al., 2012).

Colorectal neoplasia differentially expressed potentiates glioma development possibly by maintaining the stemness of the tumor cells, as it functions in neural precursors (Ellis et al., 2012; Watkins and Sontheimer, 2012). In support of this notion, a previous study by Zheng J. et al. (2015) demonstrated a direct link between the overexpression of CRNDE and GSCs. Mechanistically, CRNDE could negatively regulate miR-186 and depress the expression of the downstream target genes XIAP (X-linked inhibitor of apoptosis) and PAK7 [p21 protein (Cdc42/Rac)-activated kinase 7], thus contributing to the malignant characteristics of human GSCs (Zheng J. et al., 2015). In addition to these observations, Wang Y. et al. (2015) showed that the overexpression of the CRNDE transcript promotes glioma cell growth *in vitro* and *in vivo* through mammalian target of rapamycin (mTOR) signaling. Intriguingly, epigenetic modifications, including histone acetylation in the promoter region, can also promote CRNDE expression (Wang Y. et al., 2015). More recently, CRNDE was reported to promote malignant behavior by attenuating the miR-384/PIWIL4 (piwi-like RNA-mediated gene silencing 4) axis. Briefly, CRNDE knockdown can decrease the protein level of PIWIL4, a target of miR-384, which leads to glioma regression *in vivo* (Zheng J. et al., 2016). Overall, these results revealed that CRNDE could potentiate glioma via multiple signaling pathways and may be a promising novel therapeutic target for glioma therapy.

### LncRNA CASC2

Cancer susceptibility candidate 2, located at chromosome 10q26, is a lncRNA originally identified as a tumor suppressor gene in endometrial cancer. CASC2 consists of three alternatively spliced transcript isoforms, CASC2a, CASC2b and CASC2c, which contain identical first three exons and diverse downstream exons (Zhao et al., 2014). CASC2a expression is down-regulated at the transcription level in endometrial cancer. Baldinu et al. (2007) revealed that the exogenous expression of CASC2a in undifferentiated endometrial cancer cells significantly inhibits the clonal growth. Using a positional candidate approach, 7% CASC2a mutations in tumor DNA from 44 endometrial cancer patients were identified (Baldinu et al., 2004), suggesting that inactivation of CASC2a might probably be due to mechanisms different from genetic alterations. In non-small cell lung cancer (NSCLC) tissues and cell lines, He X. et al. (2016)) reported that CASC2 expression is involved in the development and progression of NSCLC. However, little is known about the role and function of CASC2 in human gliomas.

Recently, Wang P. et al. (2015) reported that CASC2 expression is decreased in glioma tissues as well as glioma cell lines (U251 and U87). Consistent with previous studies in other tumors, the overexpression of CASC2 could inhibit the malignancy of glioma cells through an arrest of proliferation and migration, correspondingly promoting cellular apoptosis. RIP and RNA pull-down assays confirmed that the tumor suppressive role of CASC2 is mainly mediated via the down-regulation of miR-21, one potential direct target of CASC2, in a sequence-specific manner (Wang P. et al., 2015). A growing body of literature has shown that miR-21 serves as an oncogene, and the inhibition of miR-21 is a novel therapeutic strategy for specific and effective action against gliomas (Harmalkar et al., 2015; Belter et al., 2016). Mechanistically, miR-21 promotes gliomagenesis by regulating multiple oncogenesis-related processes, including proliferation, apoptosis, migration and invasion. Therefore, targeting the CASC2-miR-21 axis may be an effective strategy for the treatment of malignant gliomas.

### LncRNA XIST

X-chromosome inactivation (XCI) ensures dosage compensation between the sexes in mammals and is a paradigm for allele-specific gene expression on a chromosome-wide scale. The lncRNA XIST, a product of the XIST gene, is located within the 500 kb stretch of XCI DNA at Xq13, which is known as the X-inactivation center (XIC); XIST is the master regulator of X chromosome inactivation in mammals (Furlan and Rougeulle, 2016; Maduro et al., 2016). The current model proposes that XIST induces epigenetic silencing of multiple genes by recruiting the chromatin modifier, the PRC2 complex, to the XIC (Goodrich et al., 2016). With the help of the high-affinity RNA-binding protein ATRX (alpha thalassemia/mental retardation syndrome X-linked), a growing number of XIST RNAs accumulate and are tethered to the X chromosome. Afterward, the XIST RNA spreads and forms a RNA “cloud” coating the XIC in *cis*. To recruit PRC2, the XIST RNA first associates with approximately 150 intense PRC2 binding sites (CpG islands), followed by its association with 3,000–4,000 moderate-strength binding sites of PRC2. Finally, XIST RNA spreads to both gene-rich and poor regions in distinct stage-specific forms on the X chromosome (Simon et al., 2013; Sarma et al., 2014).

X-inactive specific transcript has been found to be dysregulated in a variety of human cancers (Yildirim et al., 2013; Tantai et al., 2015). Specifically, a recent study showed that XIST expression is abnormally up-regulated in glioma tissues and GSCs. The knockdown of XIST by short-hairpin RNA exerts a tumor suppressive function in GSCs. Furthermore, as XIST and miR-152 may form a reciprocal repression feedback loop and are located in the same RNA induced silencing complex (RISC), miR-152 can mediate the promotion of GSCs by XIST (Yao et al., 2015). In addition, XIST can inhibit hepatoma cell proliferation and metastasis by targeting miR-92b (Zhuang et al., 2016). Moreover, XIST has been identified to directly bind tomiR-210 (Fasanaro et al., 2009). Consistently, other miRNAs, such as miR-92b and miR-210, may also regulate the expression of XIST in gliomas. Altogether, further studies should focus on the XIST-miRNA axis in glioma research and treatment.

### LncRNA TUG1

Taurine up-regulated gene 1, a 7.1 kb lncRNA located at chromosome 22q12, is a cancer-related lncRNA in some tumors, including NSCLC (Zhang et al., 2014), bladder cancer (Tan et al., 2015) and gliomas (Li J. et al., 2016). TUG1 was first identified in a genomic screen for genes differentially regulated by taurine in developing mouse retinal cells. Furthermore, TUG1 is found to play crucial roles in the formation of photoreceptors and retinal development (Young et al., 2005).

Recent investigations have reported that in human glioma cell lines, TUG1 is down-regulated, in response to necrosis induced by a high dose of DOX (Liu Q. et al., 2015). Li J. et al. (2016) showed that TUG1 acts as a tumor suppressor in glioma tumorigenesis, and is negative correlated with glioma grade, tumor size, and overall survival. Further studies via gain- and loss-of-function assays revealed that TUG1 induces glioma cell apoptosis through caspases-mediated intrinsic pathways, rather than the Bcl-2-mediated anti-apoptotic pathway (Li J. et al., 2016). However, the precise mechanism of TUG1 in cell proliferation, as well as invasion, in glioma development is still unclear. The BTB limits the effect of conventional chemotherapy by restricting drug delivery to brain tumor tissues (Hendricks et al., 2015). Using a co-culture assay with glioma and endothelial cells, Cai et al. (2015) revealed that the knockdown of TUG1 could reduce tight junction protein expression in endothelial cells by down-regulating heat shock transcription factor 2 (HSF2), the target of miR-144, increasing BTB permeability of chemotherapeutic agents. Thus, there may be potential role of TUG1 in anti-glioma therapy, and BTB function may represent a useful therapeutic intervention strategy in the future.

### LncRNA NEAT1/2

Nuclear enriched abundant transcript 1 (NEAT1) is an essential lncRNA for the formation of paraspeckles, which are nuclear bodies named for their close proximity to nuclear speckles (Yu and Shan, 2016). NEAT1 is an unusual RNA polymerase II (pol II) transcript that lacks introns, and it is widely expressed in many types of mammalian cells (Naganuma and Hirose, 2013). NEAT2/MALAT1 is a highly conserved lncRNA associated with tumorigenesis and plays a prognostic role in various cancers (Wei and Niu, 2015).

Up to date, lncRNAs have been demonstrated to be involved in the DNA damage response, thus contributing to the process of cellular defense against genotoxic agents (Zhang and Peng, 2015). Upon treatment with the DNA damage-inducing agent resveratrol, NEAT1 is up-regulated in the glioma cell lines U251 and U87 (Liu Q. et al., 2015). An increase in NEAT1 expression has also been reported in human glioma tissues compared with non-cancerous brain tissues. NEAT1 promotes glioma pathogenesis by regulating glioma cell proliferation, invasion, and migration. Zhen et al. (2016) demonstrated that functioning as a molecular sponge for miR-449b-5p, NEAT1 could up-regulate the expression of c-Met, a direct target of miR-449b-5p, thus promoting glioma oncogenesis. Furthermore, clinical investigations revealed that aberrant NEAT1 expression is negatively associated with clinical outcome in high-grade glioma patients (He C. et al., 2016).

Recent works have illustrated the tumor-suppressive role of NEAT2 in the development of glioma cells. NEAT2 expression is lower in glioma tissues than in normal brain tissues. Mechanistically, NEAT2 inhibits the proliferation and invasion of glioma cells (U87 and U251) by inactivating ERK/MAPK signaling and down-regulating MMP2 (matrix metalloproteinase 2; Han et al., 2016a). In contrast, Xiang et al. (2016) showed an opposite role of NEAT2 in gliomas. According to their observations, NEAT2 expression is significantly increased in glioma tissues, as well as in U87 and U251 cells (Xiang et al., 2016). Remarkably, GSCs of the U87, SHG44 and SHG139 cell lines expressed higher levels of NEAT2 than their parental lines (Han et al., 2016a). In addition, Han et al. (2016b) found that the down-regulation of NEAT2 suppresses the expression of stemness markers Sox2 and Nestin in SHG139S cells, while NEAT2 down-regulation promotes the proliferation of SHG139S cells. Therefore, NEAT2 plays a complex role in gliomagenesis as both a positive and a negative regulator, possibly based on its specific cellular context.

### LncRNA GAS5

Growth arrest-specific 5, localized at chromosome 1q25.1, could transcribe a tumor-suppressive lncRNA in human cancers. To date, GAS5 has been considered to act as a “riborepressor” or “miRNA sponge” that modulates the transcriptional activity of cancer-associated genes (Kino et al., 2010; Zhang Z. et al., 2013). Recent studies have reported that GAS5 negatively regulates the growth of cancer cell lines *in vitro* and *in vivo*, including gliomas (Pickard and Williams, 2015). GAS5 exerts complementary effects on cell proliferation (inhibitory) and apoptosis (stimulatory), and taken together, these cellular mechanisms likely form the basis of its tumor-suppression action (Yin et al., 2014; Shi X. et al., 2015). Mechanistically, the up-regulation of Gas5 increases the expression of tumor suppressor bmf (Bcl-2-modifying factor) and Plexin C1 via directly reducing the expression of miR-222 (Zhao X. et al., 2015). In addition, the overexpression of GAS5 could enhance the cellular response to erlotinib, a tyrosine kinase inhibitor used as a second line treatment for glioma (Garcia-Claver et al., 2013). The induction of GAS5 is apparently detected during DOX-induced apoptosis in human glioma cell lines (Liu Q. et al., 2015). The above examples suggest that GAS5 may be used as diagnostic markers or therapeutic targets for gliomas, but much work needs to be done before such applications become clinically practical.

### LncRNA ADAMTS9-AS2

The ADAMTS family has been implicated in essential physiological processes, such as angiogenesis and organ development (Ho et al., 2016). ADAMTS9-AS2 is the antisense transcript of ADAMTS9, a member of the ADAMTS family. Walsh et al. (2016) pointed that ADAMTS9-AS2 plays a critical role in epigenetic regulation, affecting early stage digit development. Recently, the ADAMTS9-AS2 locus has been revealed as a potential therapeutic target and prognostic marker in gliomas. ADAMTS9-AS2 serves as a tumor suppressor, which is significantly down-regulated in glioma tissues, and its expression is negatively correlated with tumor grade and prognosis. Meanwhile, DNMT1 knockdown remarkably enhances ADAMTS9-AS2 expression, inhibiting cell migration in gliomas (Yao et al., 2014).

### LncRNA SPRY4-IT1

SPRY4-IT1, a 708 bp intron-retained lncRNA localized at chromosome 5q31.3, is found to be significantly expressed in breast cancer (Shi Y. et al., 2015), osteosarcoma (Ru et al., 2016) and bladder cancer (Zhao X.L. et al., 2015), and its suppression can inhibit proliferation and induce apoptosis in cancer cells. SPRY4-IT1 was originally reported by Khaitan et al. (2011) to play an important role in the molecular etiology, modulation of cell apoptosis and invasion of human melanoma. Recently, the expression of SPRY4-IT1 is shown to be significantly expressed in glioma tissues and glioma cell lines compared with normal donors (Liu H. et al., 2015). The epithelial-to-mesenchymal transition (EMT), as a relevant molecular event in malignant gliomas, is an essential process in tumor dissemination and metastatic behavior (Kahlert et al., 2013). Liu H. et al. (2015) showed that the knockdown of SPRY4-IT1 by siRNA could suppress the EMT phenotype in glioma cells (U251 and SF295). However, the exact mechanism underlying the role of SPRY4-IT1 in glioma pathology still remains to be elucidated.

### LncRNA HULC

Highly up-regulated in liver cancer has pro-oncogenic activity in many human malignancies, such as B-cell lymphoma (Peng et al., 2016), hepatocellular carcinoma (Huang et al., 2016), and osteosarcoma (Sun et al., 2015). Recently, Zhu’s et al. (2016) reported that HULC has important biological function in human gliomas. HULC can promote the angiogenesis, one hallmark of malignant gliomas, by inhibiting the expression of angiogenesis-related molecule ESM-1 (endothelial cell specific molecule 1). In addition, the PI3K/AKT/mTOR signaling pathway is involved in the response induced by HULC (Zhu et al., 2016). These intriguing findings will help pave the way for exciting functional studies of HULC in gliomagenesis.

## Conclusion and Remarks

Long non-coding RNA-based mechanisms alter cell fate during development, and their dysregulation underscores many human disorders, including gliomas. LncRNAs play indispensable roles in the onset and progression of this malignancy, including the proliferation, metastasis and EMT of glioma cells. Though previously considered “junk sequences” in our genomes, the epigenetic role of lncRNA should promise to be another exciting marker for glioma research and therapy. In addition, extracellular vesicles (EVs), like exosomes, isolated from blood, cerebrospinal fluid (CSF), and other biofluids of GBM patients could offer new insight into cancer biology with both diagnostic and therapeutic implications. These exosomes have been found to harbor glioma-derived specific lncRNAs that are significantly different in cancer patients compared with normal controls (Chistiakov and Chekhonin, 2014). Moreover, exosome-transmitted lncRNAs could promote chemotherapeutic resistance in cancer by acting as a competing endogenous RNA (ceRNA; Qu et al., 2016). They can act as sponges for competitively binding miRNAs through their miRNA-recognizing elements (MREs) and further regulate the expression of miRNAs (Denzler et al., 2014; Yang et al., 2016). Strikingly, these MRE elements implicated in the ceRNA networks are also able to regulate the mRNA expression playing critical roles in tumorigenesis (Guo et al., 2015). Understanding the key roles of “lncRNA-miRNA” and “lncRNA-mRNA” interactions in the pathogenesis of gliomas will lead to the identification of new targets for GBM treatment.

In addition, TMZ, an alkylating agent, is the most widely used and effective first-line chemotherapeutic drug for treating primary and recurrent high-grade gliomas (Messaoudi et al., 2015). TMZ could activate autophagy in tumor cells. Autophagic modulators could lead to either cell survival or cell death, depending on the cellular context, which further affects the therapeutic sensitivity of TMZ in GBM (Yan et al., 2016). Recently, it has been proposed that serving as factors in gene regulation, lncRNAs could control cellular processes such as autophagy in disease conditions (Choudhry et al., 2016). The oncogene lncRNA HNF1A-AS1 could promote tumor growth by sponging tumor-suppressive hsa-miR-30b-5p in hepatocellular carcinoma. Meanwhile, the HNF1A-AS1-miR-30b axis could significantly up-regulate cell autophagy during starvation by enhancing the expression of ATG5, the target of miR-30b (Liu Z. et al., 2016). However, upon energy stress, lncRNA NBR2 (neighbor of BRCA1 gene 2) could promote AMP-activated protein kinase (AMPK) activity through interacting with AMPK, leading to a depressed autophagy response and increased tumor development (Liu X. et al., 2016). Thus, further investigation of lncRNAs in autophagy regulation would be able to identify novel strategies to enhance the benefits of TMZ chemosensitivity and chemoprotection in the treatment of gliomas.

In the last decade, lncRNAs have been regarded as molecular targets for the treatment of many cancers, including gliomas (Lavorgna et al., 2016). Furthermore, recent advancements in deep sequencing are now providing new tools to functionally annotate disease-associated lncRNAs, facilitating the identification of these new transcripts for cancer therapy (Huarte, 2015; Zheng L.L. et al., 2016). However, their biological effects are easily influenced by many factors, such as delivery strategies to cross the BTB. A better understanding of the real efficacy and mechanisms of lncRNAs, particularly in human patients, represents a matter of great interest for possible clinical application in future. Ma et al. (2016) found that the knockdown of the lncRNA NEAT2 in gliomas could result in the significantly increased permeability of BTB, which might contribute to enhancing potential therapeutic strategies for human gliomas. Meanwhile, the results from Liu’s group indicated that the lncRNA TUG1, which is highly expressed in vascular endothelial cells from glioma tissues, could influence BTB permeability via binding to miR-144, further reducing the expression of tight junction proteins in endothelial cells, such as ZO-1, occludin, and claudin-5 (Cai et al., 2015). Thus, extensive work should focus on the role of lncRNAs in BTB permeability, which may represent a useful therapeutic target for human glioma treatment.

## Author Contributions

YY, ZX and ZL wrote this review article. LS and ZG designed the study and contributed in manuscript preparation.

## Conflict of Interest Statement

The authors declare that the research was conducted in the absence of any commercial or financial relationships that could be construed as a potential conflict of interest.

## Abbreviations

ADAMTS: a disintegrin and metalloproteinase with thrombospondin motif
BTB: blood-tumor barrier
CASC2: cancer susceptibility candidate 2
CRNDE: colorectal neoplasia differentially expressed
CSCs: cancer stem cells
GAS5: growth arrest-specific 5
GBM: glioblastoma
HOTAIR: Hox transcript antisense intergenic RNA
HULC: highly up-regulated in liver cancer
lncRNAs: long non-coding RNAs
NEAT1/2: nuclear enriched abundant transcript ½
TMZ: temozolomide
TUG1: taurine up-regulated gene 1
XIST: X-inactive specific transcript
